# DMPE-PEG scaffold binding with TGF-β1 receptor enhances cardiomyogenic differentiation of adipose-derived stem cells

**DOI:** 10.1186/s13287-018-1090-z

**Published:** 2018-12-29

**Authors:** Fei Zhang, Yuan Xie, Yuhao Bian

**Affiliations:** 1 Department of Cardiothoracic Surgery, The 6th Affiliated Hospital of Shenzhen University Health Science Center, No. 89, Taoyuan Road, Nanshan District, Shenzhen, 518000 Guangdong China; 2 Xiangya School of Medicine, Central South University, Changsha, 410200 Hunan China

**Keywords:** DMPE-PEG, hADSCs, rTGF-β1 RI, TGF-β1, Myocardial tissue engineering

## Abstract

**Background:**

Heart failure has become a global health problem with increasing incidences worldwide. Traditional pharmacological treatments can delay but cannot reverse the underlying disease processes. The clinical application of myocardial tissue engineering represents a promising strategy because it features cell-based replacement therapies that replace partially or fully damaged cardiac tissues with in vitro-generated tissue equivalents. However, the effectiveness of this therapy is limited by poor viability and differentiation of the grafted cells. This limitation could be overcome by rapidly increasing the numbers of functional cardiomyocytes. In this study, we aimed to obtain functional myocardial tissue engineering seed cells with high proliferation and differentiation rates by combining 1,2-dimyristoyl-sn-glycero-3-phosphoethan-olamine-polyethylene glycol (DMPE-PEG) and recombinant transforming growth factor-β1 receptor I (rTGF-β1 RI), followed by binding to human adipose-derived stromal cells (hADSCs).

**Methods:**

To induce higher expression level of TGF-β1 RI, DMPE-PEG was inoculated with rTGF-β1 RI to modify the surface of hADSCs. The differentiation ability and morphological characteristics of the modified hADSCs were examined in vitro and in vivo.

**Results:**

The caridiomyocartic differentiation ability of TGF-β1 RI-modified hADSCs was significantly enhanced, as indicated by elevated expression levels of the cardiac markers cardiac troponin T (cTnT) and α-smooth muscle actin (SMA) via increased phosphorylation of the Smad signaling pathway-related proteins.

**Conclusion:**

Our findings provide new insights into stem cell transplantation therapy in myocardial tissue engineering.

## Background

With increasing incidences worldwide, heart failure has become a major global health issue. The heart is a complex organ composed of various cell types, including cardiomyocytes and nonmyocytes, which consist of fibroblasts, vascular cells, and inflammatory cells. According a study that analyzed myocardial cell population using fluorescence-activated cell sorting, the adult murine heart consists of ≈ 56% myocytes (cardiac troponin T cTnT+), 27% fibroblasts (discoidin domain receptor 2+), 7% endothelial cells (CD31+), and 10% vascular smooth muscle cells (α-smooth muscle actin+) [1], although the percentages might differ according to the markers used for labeling each cell type. Therefore, cardiomyocytes represent highly specific lineages, as they are the main cell lineages of the heart tissues. For patients with end-stage ischemic heart diseases, the death of numerous functioning cardiomyocytes is the leading cause of the deterioration of cardiac function [2]. In recent decades, the strategies aiming to increase the numbers of functioning cardiomyocytes have attracted great attention, as adult cardiomyocytes are regarded as terminal differentiation cells that do not differentiate or at the very least have an extremely low differentiation ability [3].

With the rapid developments of in vitro cell culture and tissue engineering technologies, myocardial tissue engineering provides new and exciting possibilities for the treatment of end-stage ischemic heart disease. The application of myocardial tissue engineering in end-stage ischemic heart diseases begins with the transplantation of cardiomyocytes [4]. The in situ construction of engineered myocardial tissue features a strategy where functional seed cells, such as cardiac muscle stem cells, bone marrow mesenchymal stem cells, and induced pluripotent stem cells, are transplanted into the heart together with bioactive molecules (including growth factors and drugs) in order to repair or replace damaged myocardial tissues [5]. The success of myocardial tissue engineering relies heavily on the seed cells, which should possess certain indispensible qualities. First, the cells should be easy to access, culture, and proliferate. Second, the cells should have no or very low immunogenicity. Finally, the cells should have the potential to differentiate into mature functional cardiomyocytes both in vivo and in vitro [6].

Great efforts of researchers have been focused on assessing the differentiation ability of various stem cells types with the aim to find the optimal seed cells for myocardial tissue engineering. Adult stem cells, especially the bone marrow mesenchymal stem (BMSC) cells, exhibited both high proliferation and differentiation potentials in vitro and were considered promising seed cells for tissue engineering. However, the differentiation potential of BMSC has been recently questioned [7, 8]. Whether BMSC can be used as seed cells for cardiomyocyte tissue engineering and transplantation remains to be determined.

Adipose stem cells (ADSCs) have held the promise of being valuable seed cells in myocardial tissue engineering since been first discovered in human adipose tissue by Hedrick in 2001 [9, 10]. There are several advantages associated with using ADSCs as myocardial seed cells in place of BMSCs. ADSCs are in great abundance and can be easily obtained from subcutaneous tissues of numerous organs. Furthermore, ADSCs are easy to isolate because of the natural buoyancy of adipocytes. In contrast, BMSCs are of low abundance in bone marrow and are mixed with many other cell types, such as prokaryotic cells, resulting in difficulties of isolation and culture. Studies have shown that specific factors can promote the differentiation of ADSCs into adipogenic, osteogenic, chondrogenic, and myocardial lineages [11]. Researchers have also demonstrated that under normal conditions, adipose stromal cells can differentiate into cells with systolic functions similar to myocardial cells. Nevertheless, the main drawback of ADSCs is that the differentiation rate is very low (0.02–0.07%) [12]. After induction with TGF-β1 for 1 or 2 weeks, ADSCs were found to stimulate the expression of cardiac systolic function-related proteins such as major histocompatibility complex (MHC), cardiac troponin (cTnT), and α-smooth muscle actin (SMA), indicating that ADSCs have potential therapeutic effects in the regeneration of infarcted myocardium. Other in vitro studies also suggest that TGF-β1 could induce myogenic differentiation of ADSCs [13]. However, myogenic differentiation of ADSCs is rare. This is most likely due to the low expression level of TGF-β1 receptor type I (TGF-β1 RI) in undifferentiated ADSCs, thus resulting in decreased sensitivity of ADSCs to TGF-β1 and diminished differentiation rate of ADSCs into myocardial cells. Therefore, to test whether ADSC differentiation capabilities are influenced by TGF-β RI expression level, the level of TGF-β RI expression needs to be modified in the cellular membrane of ADSCs. However, a major limitation is that neither DNA transfection nor viral transduction has the potential to significantly change the expression level of TGF-β RI.

Cell surface modification by natural or synthetic polymers is a new technology that holds great promise in the field of biomedical engineering. Currently, there are three categories of modification strategies: (1) hydrophobic interaction, (2) covalent conjugation, and (3) electrostatic interaction. Lipid-PEG has attracted much attention for its ability to form a uniform ultrathin coating on the surface of cells without covalent bonds or electrostatic interactions, either one of which can lead to severe cytotoxicity [14].

In this study, we aimed to generate ideal seed cells with high differentiation rate for the clinical application of cardiac regeneration tissue engineering. Using DMPE-PEG, we connected rTGF-β1 RI to the membrane of hADSCs to increase the expression of TGF-β1 RI and evaluated the differentiation ability of the modified hADSCs under exogenous TGF-β1 treatment. The results of this study provide new insight into stem cell transplantation therapy.

## Materials and methods

### Isolation and characterization of hADSCs

Ethics approval was obtained from the Ethics Committee of People’s Hospital of Nanshan District and Shenzhen University Hospital, and informed written consent was obtained from each induvial cell donor. Subcutaneous abdominal adipose tissue was obtained from women undergoing selective liposuction. Adipose tissue was immediately washed with Dulbecco’s phosphate-buffered saline (DPBS, Thermo Fisher Scientific, MA, USA), minced to small pieces and digested with 0.075% collagenase type A (Thermo Fisher Scientific, MA, USA) for 30 min at 37 °C. The digestion was terminated by α-minimal essential medium (Thermo Fisher Scientific, MA, USA) containing 10% fetal bovine serum (FBS, Thermo Fisher Scientific, MA, USA). After 10 min of centrifugation at 1200 rpm, the cell pellet was resuspended with DPBS and then filtered through a 100-μm mesh filter to remove undigested adipose tissue. The dissociated cells were seeded in a T25 culture disk (Thermo Fisher Scientific, MA, USA) at a density of 3 × 10^5^ cells/cm^2^ and cultured with α-Minimal Essential Medium containing 10% FBS and 100 U/mL penicillin/streptomycin (Thermo Fisher Scientific, MA, USA). Cells were incubated at 37 °C in a humidified atmosphere of 5% CO_2_, and the medium was changed every 3 days until the cells reached nearly 80% confluency. Cells were subcultured when they reached 80% confluency.

Immunophenotypes of hADSCs were identified by fluorescence-activated cell sorting (FACS) using specific antibodies. A total of 5 × 10^5^ cells were incubated with 10 μL fluorescein isothiocyanate (FITC, Thermo Fisher Scientific, MA, USA) conjugated mouse anti-rabbit monoclonal antibodies of CD34, CD106, CD105, and CD90 (BD Biosciences, NY, USA). The fluorescence intensity of the immunoreactive cells was measured by FACS (FACScan, BD Biosciences, NY, USA), and the data was analyzed using USES Cell Quest 6.0 software (BD Biosciences, NY, USA).

### Preparation of DMPE-PEG-FITC

DMPE-PEG-NH_2_ was purchased from Nanocs, Inc. (NY, USA). DMPE-PEG-NH_2_ was dissolved in 50 mM borate buffer (pH 8.5) to a final concentration of 1 mg/mL. FITC was dissolved in DMSO to a final concentration of 10 mg/mL. A tenfold molar excess of FITC was mixed with the DMPE-PEG-NH_2_ solution and incubated with gentle shaking for 1 h at 37 °C under dark conditions. Excess FITC was removed by a Dye Removal Column (Thermo Scientific, IL, USA). To optimize the concentration of DMPE-PEG to modify the surface membrane of hADSCs, three different concentrations of DMPE-PEG-FITC were incubated with 7.5 × 10^5^ hADSCs for 10 min at 37 °C. After incubation, the cells were harvested, washed with PBS, resuspended in PBS, and subjected to FACS analysis.

### Preparation of DMPE-PEG-rTGF-β1 RI, DMPE-PEG-rTGF-β1 RI-FITC, and cy3-TGF-β1

DMPE-PEG-maleimide was purchased from Nanocs, Inc. (NY, USA); recombinant rTGF-β1 RI and TGF-β1 were purchased from R&D Inc. (MN, USA). The recombinant rTGF-β1 RI contains ten cysteines that can react with maleimide. The rTGF-β1 RI cysteine residues are active sites for conjugation and are not involved in binding to TGF-β1. DMPE-PEG-maleimide was dissolved in PBS (pH 7.4) to a final concentration of 1 mg/mL, and rTGF-β1 RI was prepared according to the manufacturer’s instructions. DMPE-PEG-maleimide was mixed with rTGF-β1 RI (molar ratio of 10–20:1) and incubated for 2 h at room temperature. The mixed solution was then filtered using Amicon Ultra (Ultracel-10 kDa, Millipore, IL, USA) to remove impurities and stored at − 80 °C for later use. For confocal imaging and FACS analysis of rTGF-β1 RI binding efficiency, rTGF-β1 RI proteins were prelabeled with FITC according to the manufacturer’s instructions (Pierce™ FITC Antibody Labeling Kit, Thermo Fisher Scientific), and then FITC-labeled rTGF-β1 RI was incubated with DMPE-PEG-maleimide to afford a DMPE-PEG-rTGF-β1 RI-FITC solution. TGF-β1 protein was labeled with Cy3 according to the manufacturer’s instructions (Lightning-Link™ Cy3 Kit, Innova Biosciences).

### hADSC surface modification

Culture-expanded hADSCs were harvested and suspended in phenol red-free media, and 3 μg of DMPE-PEG-rTGF-β1 RI or DMPE-PEG was separately incubated with 750,000 hADSCs for 2 min at room temperature. To determine the expression level of TGF-β1 RI on modified hADSCs, 1 μg/mL FTIC-labeled rTGF-β1 RI antibody was mixed with 750,000 modified hADSCs for 1 h at room temperature. Following incubation, the cells were harvested, washed with PBS, and analyed by FACS. To explore the binding ability of rTGF-β1 RI on modified hADSCs, 750,000 hADSCs were either incubated with 3 μg of DMPE-PEG-rTGF-β1 RI-FITC or DMPE-PEG-FITC for 2 min at room temperature. Following the incubation, the cells were harvested, washed with PBS, and resuspended in PBS. The modified hADSCs were incubated with 10 ng/mL Cy3-TGF-β1 at 4 °C for 30 min on a shaker to promote the binding of TGF-β1 to rTGF-β1 RI on the surface of hADSCs. Finally, the modified cells were collected and observed under a laser scanning confocal microscope.

### Cell proliferation and adhesion

10^6^ surface-modified hADSCs were seeded on a 24-well plate (Thermo Fisher Scientific), cultured for 6 h, and hADSC cell adhesion was observed by a microscope. The CCK8 Kit (Thermo Fisher Scientific) was used to detect the viability of hADSCs. 10^5^ surface-modified hADSCs were seeded in 96-well plates (Thermo Fisher Scientific); 10 μL of the CCK-8 solution was added to each well every 2 days, and the OD value was measured at 450 nm after 4 h of incubation.

### Differentiation of modified hADSCs in vitro

10^9^ modified hADSCs were inoculated with 10 ng/mL TGF-β1 serum-free culture medium for 14 days. After 14 days, the hADSCs were harvested and blocked in a 1% BSA solution followed by overnight incubation with a primary antibody against rTGF-β1 RI, α-SMA, or cTnT (Abcam, MA, USA). The cells were then rinsed three times with PBS, incubated with fluorescently labeled secondary antibody (Abcam, MA, USA) for 1 h at room temperature, washed again in PBS, stained with DAPI, and finally sealed with an anti-quencher sealer. To avoid possible biases caused by selection of the examination fields, the samples were evaluated by three independent blind observers.

### Quantitative real-time polymerase chain reaction analysis

Total cellular RNA was extracted and purified from 10^9^ cells with a Qiagen RNeasy Mini Kit (Qiagen, MA, USA) according to the manufacturer’s instructions. Reverse transcription of 1 μg total RNA template into cDNA was carried out using the PrimeScript RT reagent Kit (Takara, Tokyo, Japan). Quantitative RT-PCR assays were performed on a 7900HT PCR system (Applied Biosystems, USA) with SYBR Premix Ex Taq™ II (Takara) according to the manufacturer’s instructions. Each sample was measured in triplicate, and the relative expression levels of mRNA were calculated using the 2^−△△Ct^ method. Primer sequences used are as follows: GAPDH (forward: CCT CCT GAA CTT GAG GCA GTT T; reverse: TGT ATT GTA ACC AGT CAT CAG CA), TGF-β1 RI (forward: GCG GCG GGT GCT TGG; reverse: TTC CTG GAA ACG ACT TGT AGG T), α-SMA (forward: AGT GAC AAC TCC ACA GAG TCA; reverse: CTT CTT GCA TCT TTA GCG CCG), and cTnT(forward: GGA GCC ACC AGA GCT ATT CC; reverse: GGT CCC AGG TCC TGA GCT AT).

### Western blotting

Cells were lysed in lysis buffer (Thermo Fisher Scientific), and protein levels were measured using the BCA method (Thermo Fisher Scientific). Fifty micrograms of proteins was separated by 10–15% SDS-PAGE gel and transferred onto polyvinylidene difluoride membranes (PVDF, Thermo Fisher Scientific) using a semi-dry blotter. The membrane was blocked with 5% BSA for 1 h and then incubated with specific primary antibodies: anti-TGF-β1, anti-α-SMA, anti-cTnT, anti-p-Smad2/3, anti-Smad2/3, or anti-actin (antibodies were all purchased from Abcam, MA, USA). Following overnight incubation, the membranes were washed with 1% TBST three times (15 min each) and incubated in horseradish peroxidase-conjugated anti-rabbit or anti-mouse IgG antibodies (Abcam) at room temperature for 50 min. Following incubation, the membranes were washed with 1% TBST three times (15 min each). The protein-antibody complexes were detected using the chemiluminescent reagent WesternBright ECL detection kit (Thermo Fisher Scientific). Relative protein levels were determined in comparison with β-actin.

### Transplantation and in vivo immunofluorescent analysis of modified hADSCs

All animal experiments were approved by the laboratory animal organization committee. Eight-week-old female Sprague-Dawley rats (Harlan-IBERICA, Spain) were selected as the research objects. The rats were anesthetized by intraperitoneal injection of pentobarbital sodium (50 mg/kg) and placed on a breathing apparatus. The skin in the front of the chest was sterilized with iodine and alcohol, and an incision of about 3 cm was made between the 4th and 5th rib on the left (lateral) side of the chest. The incision involved cutting through the first two layers of the intercostal muscles, applying a small separation followed by a blunt separation of the third intercostal muscles, opening the upper chest cavity and exposing the heart. Rats were randomly divided into two groups (*n* = 9, per group): (1) D-P (*n* = 9), received an intramyocardial injection of 80 μL of 109 DMPE-PEG-modified hADSC cells; (2) D-P-rTGF-β1 RI (*n* = 9), received an intramyocardial injection of 80 μL of 109 DMPE-PEG-rTGF-β1 RI-modified hADSC cells. The chest cavity of rats was opened immediately, the cell graft was injected into the intracardiac tissue, and the injection was completed within 5 min. One week after transplantation, the experimental rats were selected according to the experimental plan. After intraperitoneal injection of chloral hydrate, the rats were sacrificed and the hearts were removed for follow-up experiments. Immunofluorescent analysis was performed on PFA-fixed frozen heart sections to evaluate the expression of cardiac markers cTnT and α-SMA. Tissue sections were first incubated in a blocking solution containing 1% BSA in PBS for 1 h at room temperature. The specimens were then incubated with anti-cTnT or anti-α-SMA primary antibodies overnight at 4 °C. All slides were washed three times in PBS and incubated with fluorescently labeled secondary antibodies at room temperature for 1 h. Following a final wash in PBS, samples were double-stained with DAPI and then cover-slipped with an anti-fade medium. The intensity of immunostaining was evaluated by three blinded independent observers. To avoid potential biases caused by the selection of the examination fields, areas suitable for analysis were selected based on the integrity of the tissue.

### Statistical analysis

Statistical analyses were performed using the SPSS 16.0 software (IBM, USA). Comparisons between groups were calculated using a two-tailed paired Student’s *t* test, ANOVA test, and the Mann-Whitney *U* test. A *p* value of < 0.05 was considered statistically significant. GraphPad Prism version 5.0 was used for scientific graphing.

## Results

### Culture and identification of hADSCs

**Fig. 1:**
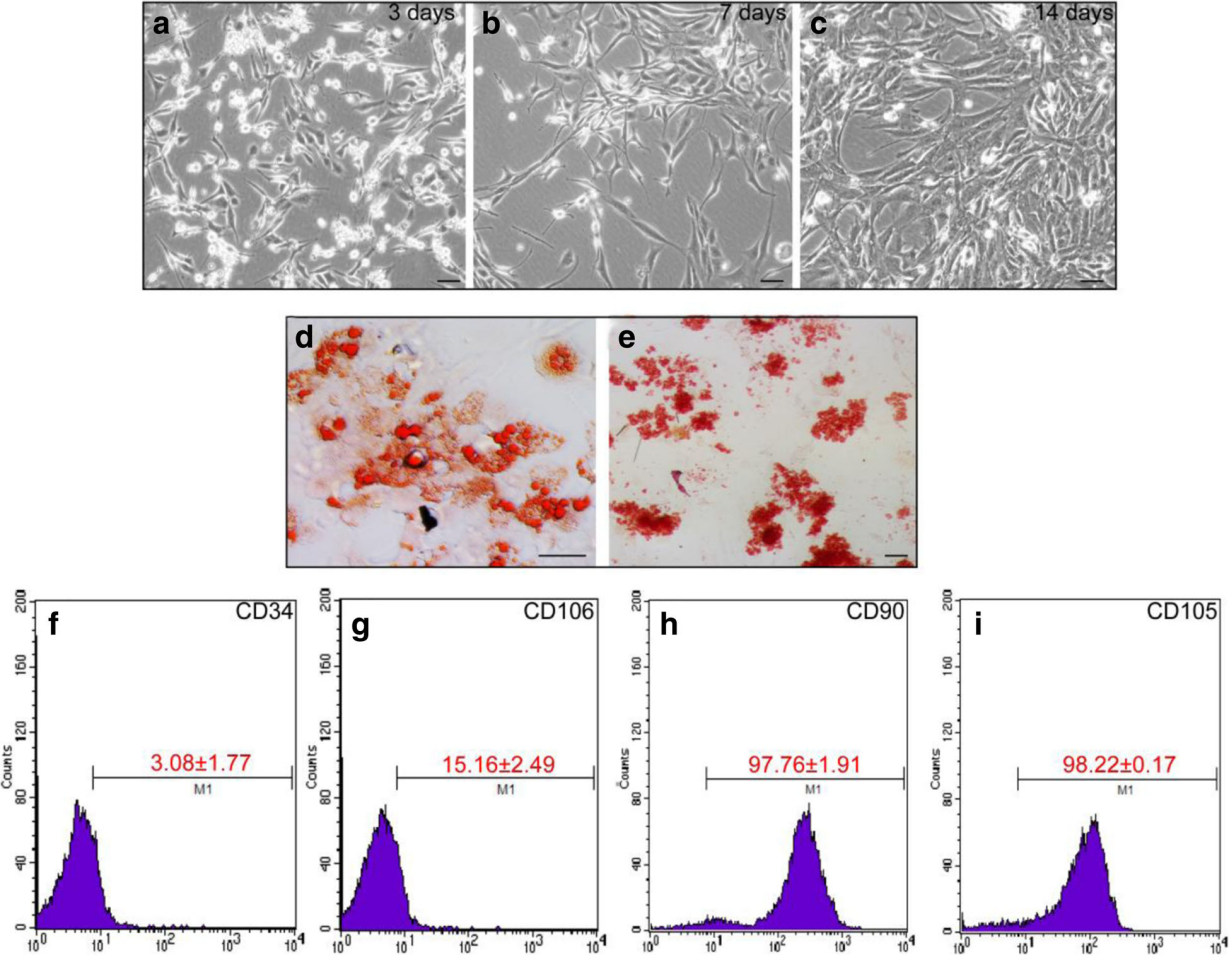
The characteristics of cultured adipose-derived mesenchymal stem cells (ADSCs) at day 3, day 7, and day 14. ADSCs were isolated and cultured from human adipocyte tissue. By day 3, most of the cells were ovoid (**a**); by day 7, fibroblast-like cells were observed (**b**); and by day 14 (**c**), the ADSCs grew into clusters with a long spindle-shaped morphology. ADSCs were cultured in a lipogenic medium, and accumulation of lipids into intracellular vesicles was observed by Oil Red O staining (**d**). ADSCs were cultured in osteogenic medium and stained with Alizarin Red S to visualize calcium deposition and mineralization in the monolayer (**e**). Flow cytometry was used to detect the expression of ADSC markers (**f**–**i**) (scale bar, 100 μm)

Based on previous literature reports, hADSCs were subsequently cultured after isolation from fat depots. Most cells were ovoid and suspended in the culture medium by day 3 (Fig. 1a) and were attached to the surface with fibroblast-like characteristics by day 7 (Fig. 1b). After 2 weeks in culture, the hADSCs grew into clusters and exhibited a long spindle shape (Fig. 1c). hADSCs derived from the mesoderm have multi-lineage differentiation potentials and can differentiate into adipocyte, osteoblast, cardiomyocyte, and other cell types. To assess the multi-differentiation potentials of cultured hADSCs, adipogenic and osteogenic differentiation were induced around 2 weeks. Oil Red O staining revealed that most of the induced hADSCs showed cytoplasmic staining of orange-red lipid droplets, consistent with the adipogenic differentiation potential of hADSCs (Fig. 1d). Meanwhile, alizarin red staining revealed that most cells contained orange-red deposits in the cytoplasm, indicating that intracellular calcium and alizarin red formed coordination complexes and that the hADSCs had differentiated into osteocytes (Fig. 1e). ADSCs derived from the mesoderm have similar differentiation potentials as mesenchymal stem cells (MSCs), but their expression levels of cell surface markers are slightly different. Interestingly, the expression of VCAM1/VLA4, the receptor-ligand pair that plays a key role in the homing and mobilization of hematopoietic stem cells, are inversely correlated in MSCs and ADSCs. While MSCs generally express VCAM1 but not VLA4, ADSCs express VLA4 but not VCAM1 (needs a reference). CD106, a component of VCAM1, has been shown to be expressed in MSCs while CD49d (a component of VLA4) is not expressed. Conversely, CD49d was expressed in ADSCs while CD106 was not [13]. The results of FACS showed that the proportions of hADSCs positive for CD34 and CD106 proteins were less than 3.08 ± 1.77% and 15.16 ± 2.49% (Fig. 1f, g), while the proportions of hADSCs expressing CD90 and CD105 were 97.16 ± 1.91% and 98.22% ± 0.17%, respectively (Fig. 1h, i). These results suggested that the hADSCs attained multi-differentiation potentials.

### Surface modification of ADSCs by DMPE-PEG

**Fig. 2:**
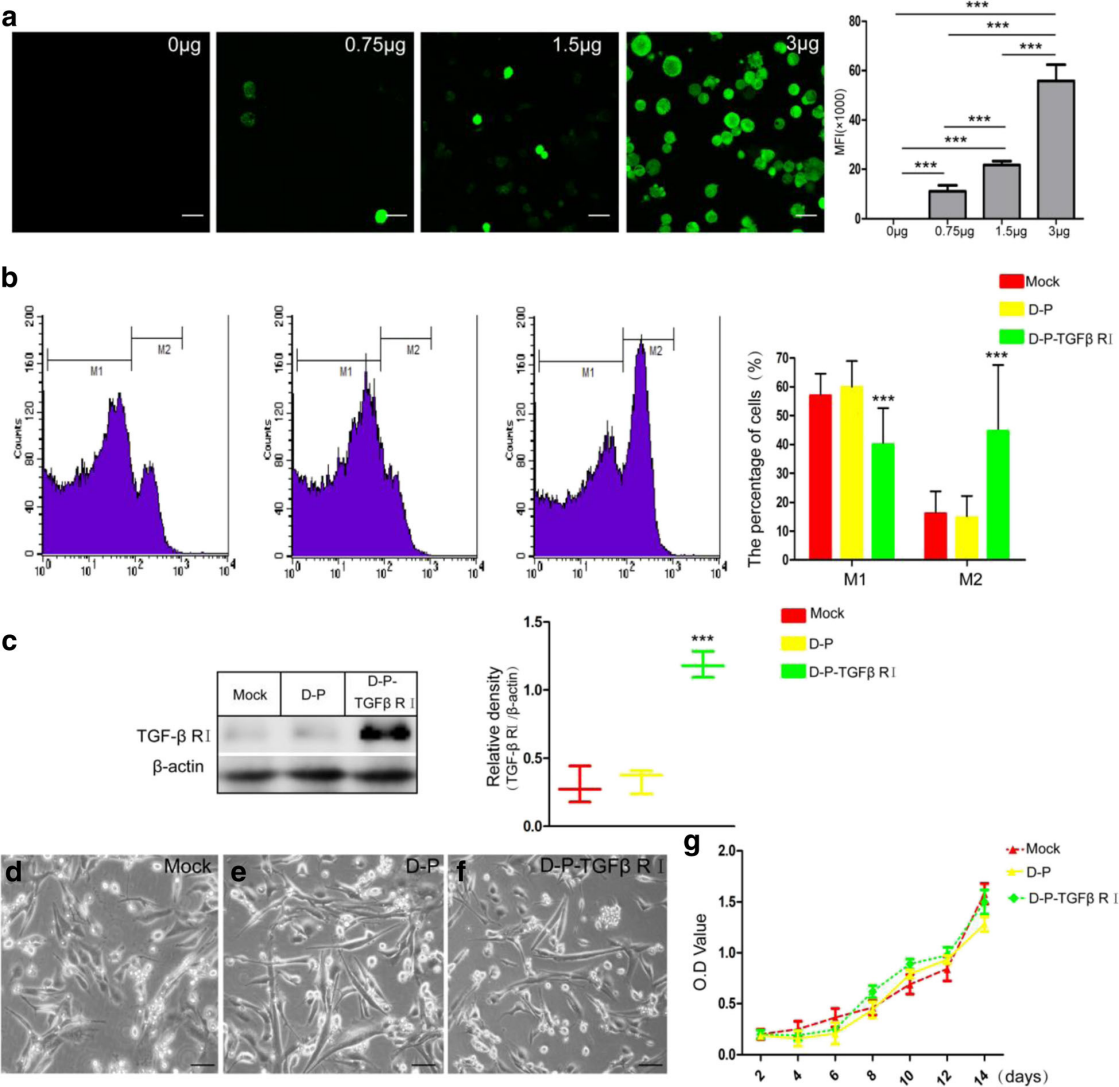
The effect of different concentrations (0, 0.75, 1.5, or 3 μg) of DMPE-PEG-FITC on ADSCs was analyzed by confocal microscope (**a**). **b** Fluorescence intensity as demonstrated by FACS analysis of TGF-β1 RI expression on the membranes of modified ADSCs. **c** Western blot analysis for the expression intensity of TGF-β1 RI protein on modified ADSCs. **d**–**f** Light microscopy analysis of the effect of DMPE-PEG on ADSCs adhesion. **g** Growth curve of modified and mock modified ADSCs (scale bar, 100 μm; *p < 0.05, **p < 0.01, ***p < 0.001)

To optimize the concentration of DMPE-PEG, different amounts (0.75, 1.5, and 3 μg) of DMPE-PEG-FITC were mixed with a fixed number of ADSCs for 2 min. After incubation, hADSCs were visualized at pre-determined time points by confocal analysis. The results showed that 750,000 hADSCs could be modified by less than 1.5 μg of DMPE (Fig. 2a). As previously reported, DMPE-PEG acted as a bridge that connected the recombinant protein to the surface of ADSCs. In order to test this hypothesis, FITC-labeled TGF-β1 RI antibody was used to detect the ADSCs surface-bridged TGF-β1 RI protein. The results of FACS revealed that 44.53 ± 23.06% ADSCs were positive for TGF-β1 RI (M2) in the DMPE-PEG-TGF-β1 RI-modified (D-P-TGFβ RI) group. In contrast, when comparing the mock group and DMPE-PEG modified (D-P) group, the fractions of TGF-β1 RI-positive hADSCs were only 16.03 ± 7.85 and 14.61 ± 7.55, respectively (Fig. 2b). Therefore, the expression level of TGF-β1 RI protein was significantly higher in the D-P-TGF-β RI group than in the mock group and D-P group (Fig. 2c). Next, we investigated the effects of cell surface modifications on hADSCs adhesion. Microscopy analyses demonstrated that no significant alteration was observed in hADSC adhesion at 6 h post-seeding (Fig. 2d–f), indicating that DMPE-PEG modification of hADSCs had no influence on the cell surface adhesion molecules. Results of proliferation assay revealed that DMPE-PEG or DMPE-PEG-rTGF-β1 RI did not negatively affect the growth of modified ADSCs, indicating that the implanted modified hADSCs could grow normally in the host myocardium (Fig. 2g). These results demonstrate that this technique could directly connect TGF-β1 RI to the cell surface and subsequently stimulate the surface expression of TGF-β1 RI without negative impacts on either cell adhesion or proliferation of hADSCs.

### Confirmation of TGF-β1 binding to rTGF-β1 RI on the surface of modified ADSCs

**Fig. 3:**
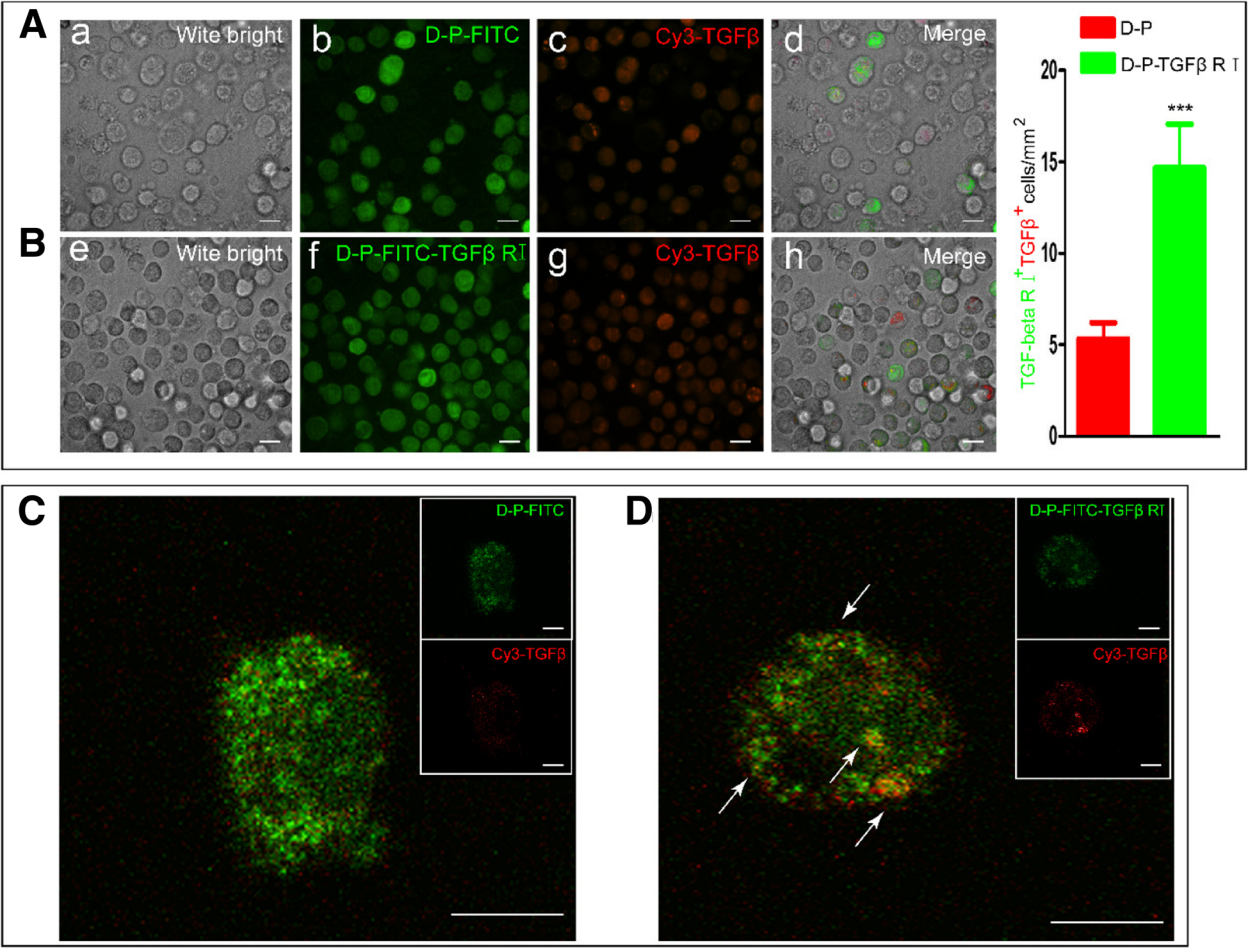
Confocal microscopy was performed to analyze (**A**, **B**) TGF-β1 binding to rTGF-β1 RI on ADSC cell membranes and to visualize (**C, D**) the colocalization of rTGF-β1 RI (DMPE-PEG-TGF-β1 RI-FITC) and TGF-β1 (Cy3-TGF-β1) on the surface of ADSCs at × 64 oil immersion (scale bar, 100 μm; *p < 0.05, **p < 0.01, ***p < 0.001)

The TGF-β1 signaling pathway regulates a variety of cellular processes, including cell proliferation, apoptosis, migration, and differentiation. TGF-β1 signaling is initiated by the binding of TGF-β1 to type II (TGF-β1 RII) and type I (TGF-β1 RI) receptors on the cell membrane. To investigate the binding interactions between rTGF-β1 RI and TGF-β1 on the surface of the hADSCs, Cy3-TGF-β1 was mixed with the modified ADSCs. Laser scanning confocal microscopy showed that strong fluorescence staining of rTGF-β1 RI-FITCand Cy3-TGF β1 was detected on the surface of most hADSCs and that rTGF-β1 RI-FITC co-localized with Cy3-TGF-β1 (Fig. 3B), indicating binding of TGF-β1 to rTGF-β1 RI on the surface of the hADSCs. In contrast, as demonstrated by fluorescent staining, relatively little Cy3-TGF-β1 was found to be co-localized with DMPE-PEG-FITC on the surface of the hADCSs (Fig. 3A). Oil immersion microscopy demonstrated that rTGF-β1 RI-FITC co-localized with Cy3-TGF-β1 to form numerous yellow dots on the surface of hADSCs modified with DMPE-PEG-rTGF-β1 RI-FITC (Fig. 3D, indicated by white arrows). However, on the surface of DMPE-PEG-FITC-modified ADSCs, red staining corresponding to Cy3-TGF-β1 was significantly reduced, and only a few yellow co-localized dots were observed (Fig. 3C, indicated by white arrows). These results confirmed that hADSCs modified by our method were able to bind to TGF-β1.

### Differentiation of surface-modified ADSCs induced by TGF-β1 in vitro

**Fig. 4:**
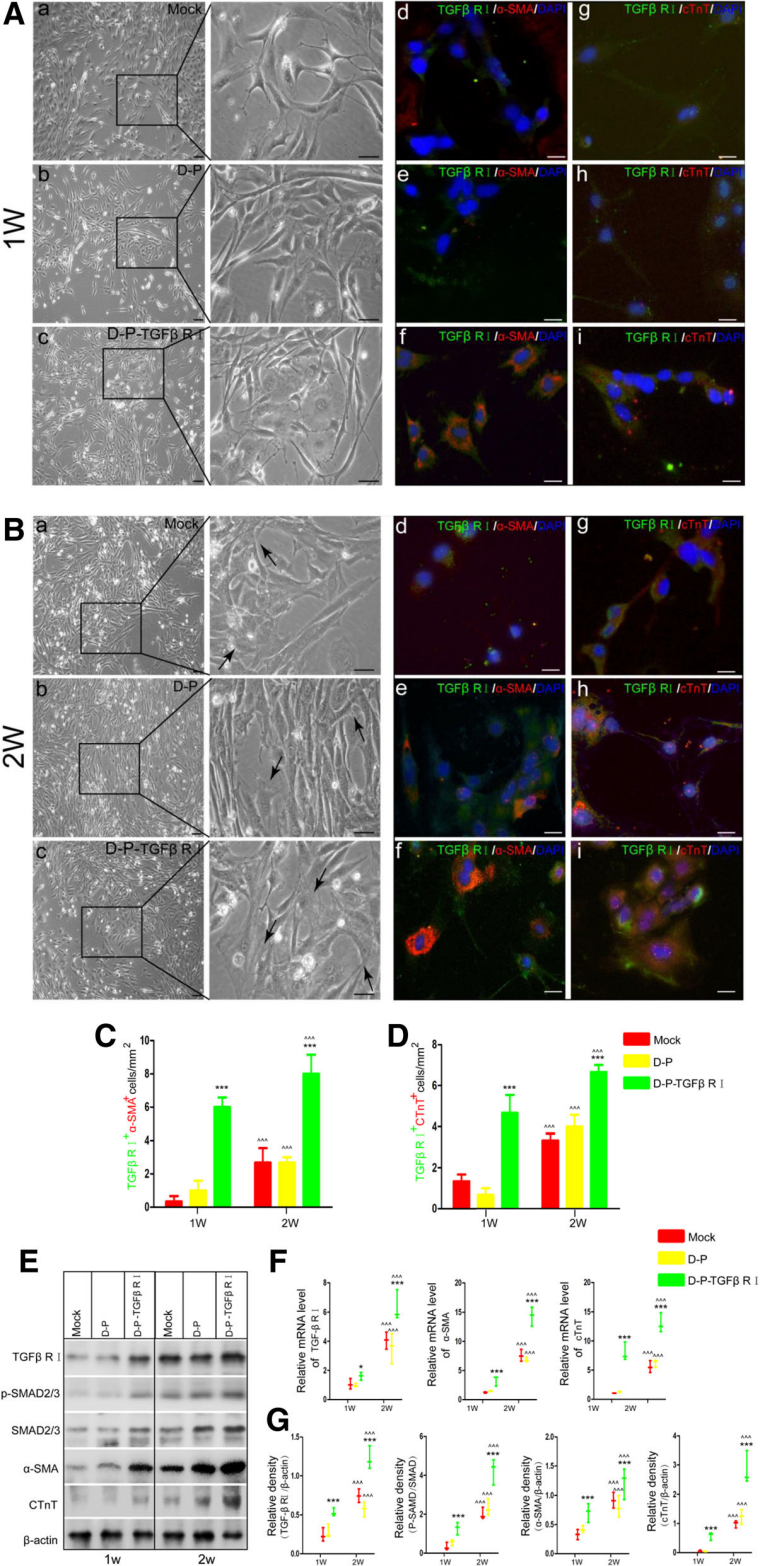
Following 1 or 2-week treatments with TGF-β1, ADSC cell morphology was compared in the mock, D-P, and D-P-TGF-β1 RI groups with a phase contrast microscope (**A**, **B**, a–c). ADSCs were immunostained with anti-TGF-β1 RI (green),anti-α-SMA (red), and cTnT (red). Nuclei were counterstained with DAPI (blue) (**A**, **B**, d–i). **C**, **D** The number of cells expressing TGF-β1 RI /α-SMA and TGF-β1 RI /cTnT were compared. **E** Western blot was performed to exam the protein expression of TGF-β1 RI, α-SMA, cTnT, p-SMad2/3, and SMad 2/3 in the mock, D-P, and D-P-TGF-β1 RI groups. **G**, **F** Comparison of relative mRNA and protein expression levels of TGF-β1 R1, α-SMA, and cTnT in the mock, D-P, and D-P-TGF-β1 RI groups (scale bar, 100 μm; values are expressed as mean ± S.D; *p < 0.05, **p < 0.01, ***p < 0.001)

Previous studies reported that ADSCs express the myocardial marker proteins α-SMA and cTnT when induced by TGF-β1 [15]. In order to further confirm the effects of TGF-β1 binding on the differentiation of modified hADSCs, the cells were treated with 10 ng/mL TGF-β1 for 2 weeks. As shown in Fig. 4A, the mock group and D-P group exhibited fibroblast-like cellular morphology after 1 week, while a portion of the D-P-TGFβ RI group was larger and exhibited the characteristic polygonal appearance of cardiomyocytes (Fig. 4A, indicated by black arrow). After 2 weeks of treatment, very few cells with cardiomyocyte morphological features were observed in the mock group or D-P group, whereas most of the TGF-β1 RI-modified hADSCs exhibited orderly actin filament arrangement in the cytoplasm (Fig. 4B, black arrows), indicating the expression of cardiomyocyte phenotypes. Furthermore, immunofluorescent staining results revealed significant increases of TGF-β1RI+ α-SMA+ cells and TGF-β1RI+ cTnT+ cells in the D-P-TGFβ RI group (6.2 ± 1.92/mm^2^, 4.8 ± 1.3/mm^2^) compared with the mock (0.8 ± 0.83/m^2^, 1.2 ± 0.84/mm^2^) or D-P (1.4 ± 1.1/m^2^, 0.8 ± 0.55/mm^2^) groups. After 2 weeks of treatment, 7.8 ± 1.43/mm^2^ of TGF-β1RI+ α-SMA+ cells and 6.8 ± 1.48/mm^2^ of TGF-β1RI+ cTnT+ cells were observed in the D-P-TGFβ RI group. These were much higher numbers than that observed in the mock (TGF-β1+ α-SMA+: 3.0 ± 1.85/mm^2^, TGF-β1+ cTnT+: 3.2 ± 1.87/mm^2^) or D-P (TGF-β1+ α-SMA+: 3.2 ± 0.837/mm^2^, TGF-β1+ cTnT+: 3.6 ± 0.55/mm^2^) groups (Fig. 4C, D). RT-PCR results demonstrated that endogenous TGF-β1RI, α-SMA, and cTnT levels were significantly elevated in the D-P-TGFβ RI group when compared with the other treatment groups at 1 and 2 weeks (Fig. 4F). The increases in expression of TGF-β1 RI, α-SMA, and cTnT in the D-P-TGFβ RI group were further confirmed by Western blot (Fig. 4E, G).

Previous studies have shown that TGF-β1 can activate the Smad2/3 signaling pathway to upregulate the expression of TGF-β1 RI on hADSCs. Our results indicated that enhancing the binding of TGF-β1 on hADSCs increased the phosphorylation level of Smad2/3, subsequently promoted the expression of myocardial marker proteins α-SMA and cTnT and eventually upregulated the expression of TGF-β1 RI. Thus, this technique established a positive feedback loop in TGF-β1 RI-modified hADSCs. These results showed that increasing the abundance of TGF-β1 RI on the cell membrane of ADSCs can improve the cellular sensitivity to TGF-β1 induction, which promotes the differentiation of hADSCs by enhancing Smad2/3 phosphorylation.

### Differentiation of surface-modified hADSCs induced by TGF-β1 in vivo

**Fig. 5:**
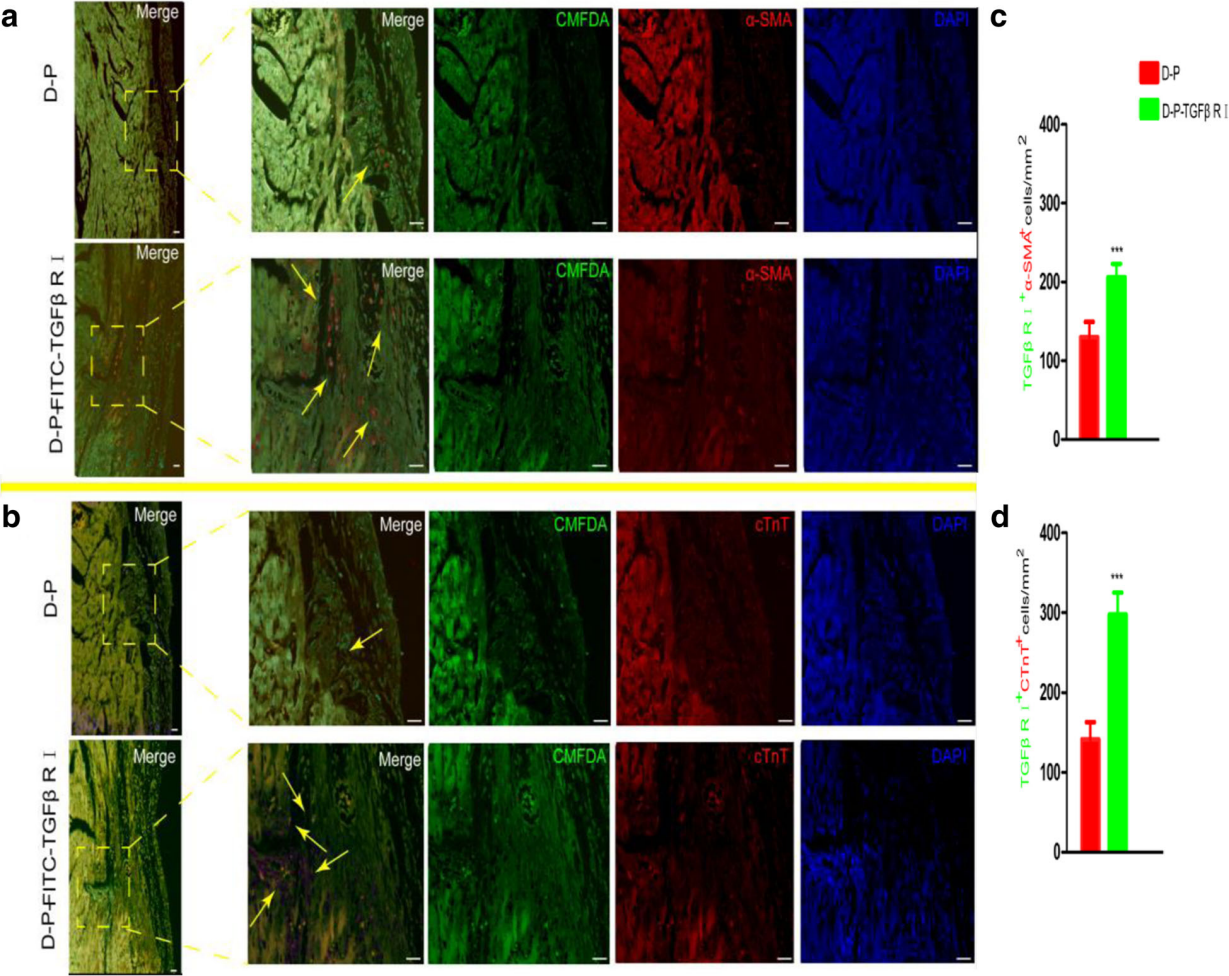
In vivo differentiation studies and immunofluorescence analysis of D-P and D-P-TGF-β1 RI samples (× 40 magnification) after 1 week of transplantation. **a**, **b** Modified ADSCs were labeled with the cell track reagent CMFDA (green) and immunostained with anti-α-SMA(red) or anti-cTnT (red). Nuclei were counterstained with DAPI (blue). **c**, **d** The number of modified ADSCs expressing α-SMA and cTnT were compared (scale bar, 100 μm; values are expressed as mean ± S.D; *p < 0.05, **p < 0.01, ***p < 0.001)

To further explore the differentiation ability of the modified cells, we used animal models to analyze the cardiomyogenic differentiation of the modified cells in vivo. One week after intramyocardial injection of the modified ADSC cells, the transplanted cells (green) could be clearly distinguished from host cells in the heart. In the D-P-TGFβ RI group, 69.96 ± 4.3% and 54.82 ± 4.29% of the transplanted cells expressed α-SMA (Fig. 5a, c) and cTnT (Fig. 5b, d), respectively. Whereas in the D-P group, only 20.45 ± 6.87% and 14.69 ± 2.76% of the transplanted cells expressed α-SMA and cTnT, respectively. Therefore, the results indicate that the hADSCs with a higher surface abundance of rTGF-β1 RI could be more easily induced into cardiomyocyte-like cells than control groups in vivo.

## Discussion

Unlike most tissues, adipose tissue is highly malleable in adults due to the hypertrophy of adipocytes. Previous studies suggest that the proliferative hypertrophy of adipocytes results from the existence of a special type of progenitor cell, namely the pre-adipocyte [16]. Additionally, researchers have demonstrated that there indeed exists a type of adipose-derived stem cells (ADSCs), which has strong regeneration and differentiation potentials in vitro. Recently, ADSCs have been widely used as a research tool because they can be readily obtained by minimally invasive surgery and differentiate into different cell types under appropriate conditions, including adipocytes, osteoblasts, cardiomyocytes, and endothelial cells [9, 17].

TGF-β1 is a multifunctional cytokine involved in various pathological and physiological processes such as the differentiation, growth, and cell survival [13]. TGF-β1 has also been shown to induce differentiation of ADSCs into myocardial cells. In this process, TGF-β1 activates the serine/threonine kinase domains of the TGF-β1 receptors, which are composed of type I and type II receptor subunits (TGF-β1 RI and TGF-β1 RII) [13]. The activated TGF-β1 receptors recruit and phosphorylate Smad2/3 proteins [18]. Following phosphorylation, Smad2 and Smad3 combine with Smad4 to form a transcription complex, which enters the nucleus and activate the transcription of target genes [19]. The present study suggests that TGF-β1 could induce ADSCs to differentiate into myocardial cells in vitro. After treatment with TGF-β1 for 1 or 2 weeks, ADSCs expressed high levels of cTnI and α-SMA proteins, accompanied by an increased rate of differentiation to myocardial cells. Expression of these proteins may be related to the slow peristalsis and contraction of the early embryo heart, thus inhibiting the differentiation of ADSCs into immature cardiac cells. Most importantly, differentiated ADSCs with high expression levels of these proteins lack the ability to differentiate into other accompanying cell lineages, such as the osteogenic linear [20].

The TGF-β1-Smad2/3 pathway is a very important signaling cascade for the differentiation of stem cells. Previous studies report that during TGF-β1-regulated differentiation of ADSCs, no correlation exists between the chondrogenic differentiation potential of ADSCs and the expression level of TGF-β1 RII in the membrane [21]. Other studies have demonstrated that the myocardial differentiation potential of ADSCs is closely related to the expression of TGF-β1 RI (needs references). However, the abundance of TGF-β1 RI on the membrane of ADSCs is significantly lower than that of BMSCs [15]. Therefore, ADSCs are usually insensitive to the induction of TGF-β1.

In this study, we aimed to prepare ideal seed cells with high proliferation and differentiation potentials for the clinical application of myocardial tissue engineering. To increase the sensitivity of hADSCs to TGF-β1 intervention, we bound rTGF-β1 RI to the surface of hADSCs using DMPE-PEG as a scaffold. As a result, the phosphorylation levels of Smad signaling pathway-related proteins and the expression levels of cTnT and α-SMA were raised, indicating that the myocardial differentiation potential of hADSCs was significantly enhanced. Our results provide new insights into stem cell transplantation therapy and myocardial tissue engineering.

## Conclusion

Our results demonstrated that increasing the abundance of TGF-β1 RI on cell membrane with a DMPE-PEG scaffold raised the sensitivity of hADSCs to TGF-β1, thereby promoting the differentiation of hADSCs to myocardiocytes via enhancing Smad2/3 phosphorylation. Thus, we offer a novel approach for the generation of excellent seed cells with high myocardiocyte differentiation rates for the clinical application of regenerative cardiac tissue engineering.

## Abbreviations

ADSCs: Adipose-derived stem cells
BMSCs: Bone marrow stem cell
DAPI: 4′,6-Diamidino-2-phenylindole
DPBS: Dulbecco’s phosphate-buffered saline
FACS: Fluorescence-activated cell sorting
FBS: Fetal bovine serum
MHC: Major histocompatibility complex
MSCs: Mesenchymal stem cells
PBS: Phosphate buffer saline
TBST: Tris-buffered saline and Tween 20
TGF-β: Transforming growth factor-β
